# Self-regulation and obesity: the role of executive function and delay discounting in the prediction of weight loss

**DOI:** 10.1007/s10865-018-9940-9

**Published:** 2018-05-25

**Authors:** Fania C. M. Dassen, Katrijn Houben, Vanessa Allom, Anita Jansen

**Affiliations:** 1 Department of Clinical Psychological Science, Maastricht University, P.O. Box 616, 6200 MD Maastricht, The Netherlands; 2 School of Psychology and Speech Pathology, Curtin University, GPO Box U1987, Perth, WA 6845 Australia

**Keywords:** Weight loss, Obesity, Body Mass Index, Executive function, Delay discounting

## Abstract

Obesity rates are rising worldwide. Executive function and delay discounting have been hypothesized to play important roles in the self-regulation of behavior, and may explain variance in weight loss treatment success. First, we compared individuals with obesity (*n *= 82) to healthy weight controls (*n *= 71) on behavioral and self-report measures of executive function (working memory, inhibition and shifting) and delay discounting. Secondly, the individuals with obesity took part in a multidisciplinary weight loss program and we examined whether executive function and delay discounting predicted weight change. Individuals with obesity displayed weaker general and food-specific inhibition, and weaker self-reported executive function. Better behavioral working memory and better self-reported inhibition skills in daily life were predictive of greater weight loss. As findings are correlational, future studies should investigate the causal relationship between executive function and weight loss, and test whether intervening on executive function will lead to better prevention and treatment of obesity.

## Introduction

The worldwide prevalence of overweight and obesity is high with 37% of adults experiencing overweight or obesity (Ng et al., 2014). Being overweight places individuals at risk of cardiovascular diseases, various forms of cancer, diabetes mellitus type II and musculoskeletal disorders (Lim et al., 2013). While there may be medical causes involved, most of the variance in Body Mass Index (BMI) can be attributed to behavioral factors (Ravussin & Bogardus, 2000) that result in an energy imbalance (i.e., more energy is consumed than expended). Thus, to achieve weight loss, one needs to reduce caloric intake, increase physical activity, or do both. However, in practice, the prevention and treatment of obesity is not as straightforward. While many individuals may engage in weight loss attempts (De Ridder et al., 2014) about 80% of overweight dieters are not able to maintain their weight loss in the long run (Wing & Phelan, 2005). Given the considerable health implications of obesity, it is important to find out what determines treatment success and successful weight loss.

Maintaining a healthy weight requires self-control (Hofmann et al., 2009a, b). According to dual-process theories of self-control a healthy lifestyle depends on the balance between two competing systems: the impulsive and the reflective (Hofmann et al., 2009a, b; Strack & Deutsch, 2004). Behaviors relating to weight gain such as the overconsumption of palatable, energy-dense foods are the result of bottom-up impulses (i.e., the impulsive system) that are not sufficiently regulated via top-down cognitive control processes (i.e., the reflective system; Hofmann et al., 2009a, b; Strack & Deutsch, 2004). Executive function lies at the heart of cognitive self-control (Hofmann et al., 2012). Executive function is an umbrella term that refers to three main cognitive functions: (1) working memory—maintaining and updating relevant information; (2) inhibition—inhibiting prepotent impulses, and (3) shifting—rapidly and efficiently adapting to different situations (Miyake et al., 2000). Executive function allows for goal-directed action, and is important for planning and monitoring behavior, suppressing undesired responses, resisting temptations and creating alternatives, which are all highly relevant for engaging in and maintaining health behaviors, such as maintaining a diet in order to achieve weight loss (Dohle et al., 2018; Hall & Marteau, 2014; Hofmann et al., 2012). For example, working memory is important for the regulation of food intake by keeping long-term goals active and down regulating cravings for immediate desires (e.g. Hofmann et al., 2008) while inhibition assists in the suppression of automatic impulses to consume tasty, high-calorie foods (e.g. Guerrieri et al., 2012; Hofmann et al., 2009a, b). Shifting has been hypothesized to be important in the selection of alternative means to pursue diet goals (e.g. Dohle et al., 2018; Hofmann et al., 2012), and has been found to be predictive of the extent to which individuals translate their healthy eating intentions into eating behavior (Allan et al., 2011).

In line with dual-process accounts, weaker executive function appears to be related to elevated BMI (for reviews see Fitzpatrick et al., 2013; Prickett et al., 2015; Smith et al., 2011; Vainik et al., 2013; Yang et al., 2018). However, the associations between BMI and each individual facet of executive function are not consistently found. Regarding inhibition, while it appears that the association between obesity and weaker inhibition is quite robust (for reviews see Bartholdy et al., 2016; Lavagnino et al., 2016), some studies only found differences in performance between individuals with obesity and healthy weight controls on a food-specific inhibition task (e.g. Houben et al., 2014; Nederkoorn et al., 2012), and others found no differences in task performance (e.g. Hendrick et al., 2012; Loeber et al., 2012; though note that in the latter differences on self-report measures were significant). The association between working memory and BMI is also inconsistent. For example, some studies demonstrate a difference in working memory between individuals with obesity and those with a healthy weight (Maayan et al., 2011; Stingl et al., 2012), while other studies find no such difference (Ariza et al., 2012; Gonzales et al., 2010). Similarly, significant differences in shifting between individuals with obesity and those with a healthy weight have been found in a number of studies (e.g. Fagundo et al., 2012; Lokken et al., 2010; Maayan et al., 2011), though not in all studies (Ariza et al., 2012; Mobbs et al., 2011).

The inconsistency in these findings can perhaps be explained by the variability in the measures used to assess these constructs. There are many cognitive tasks reported to measure the domains of executive function (Miyake et al., 2000), and as a result, there is little consistency in methodology and results both within and across different domains of executive function. Thus, when studying executive function, it is imperative that a clear rationale for the selection of measures is presented (Etnier & Chang, 2009). For the current study, representative and commonly used tasks were selected: the *n*-back task (Kirchner, 1958) to measure working memory, the Stop-Signal Task (Logan et al., 1997) to measure inhibition (general and food-specific), and the Trail Making Test (Reitan, 1958) to measure shifting. In addition to cognitive performance-based tasks, a self-report measure of executive function was also included, as both types of measures appear to capture different aspects of executive function: behavioral tasks seem to measure the efficiency of cognitive abilities while self-report measures seem more related to goal achievement in daily life (Toplak et al., 2013).

Most studies on the relationship between executive function and BMI are cross-sectional in nature and examine differences between individuals of different weights. Examining whether executive function prospectively predicts weight gain and weight loss may be more useful in terms of weight loss intervention design. However, currently, few studies have examined the association between executive function and successful weight loss during a weight-loss intervention. Nederkoorn et al. (2007) showed that children with obesity who displayed weaker inhibition skills lost less weight during a multidisciplinary residential treatment for obesity. In line with this, Manasse et al. (2017) found that weaker general inhibition (though not food-specific inhibition) was associated with less weight loss during treatment. Hege et al. (2013) showed that differences in brain activity during a working memory task predicted successful weight loss during behavioral treatment, suggesting that the ability to encode or retrieve food and weight loss goals may contribute to the regulation of eating behavior. A more extensive prospective study, examining working memory, inhibition, shifting and planning, showed that poorer performance on a shifting task and more impulsive reactions on an inhibition task were associated with less weight loss after 8 weeks (Galioto et al., 2016). This study included an extensive intervention program conducted over a short period of time using a relatively small sample. Weaker executive function has also been associated with less weight loss following bariatric surgery, possibly due to less adherence to post-operative guidelines for diet and physical activity (Spitznagel et al., 2013a, b). Thus, individual differences in executive function within individuals with obesity may explain why some individuals succeed during behavioral treatment and some do not. Currently, evidence for the role of executive function in the prediction of weight loss during treatment is insufficient.

Delay discounting is another construct that could be important in weight regulation, and was therefore included in this research. Delay discounting is the decline in value of a reward according to how temporally distal that reward is. Someone with a tendency to choose immediate over delayed rewards is considered to display greater delay discounting and therefore be more impulsive (Ainslie, 1975). As described earlier, impulsivity or a lack of self-control is counteractive to the maintenance of a healthy weight. Successful weight regulation requires that someone keeps their long term health in mind and does not succumb to tempting food stimuli. However, unhealthy eating seems associated with a focus on immediate benefits, and less concern with future consequences (e.g. Dassen et al., 2015). A tendency to discount future rewards has been related to obesity (for a review see Barlow et al., 2016), though note that this association has not been consistently found (e.g. Feda et al., 2015; Nederkoorn et al., 2006). A preference for immediate rewards could reduce the success of an intervention, while the ability to delay gratification might facilitate successful weight loss and its maintenance. Evidence for the association between delay discounting and successful weight loss is limited. In a family-based obesity treatment, children who displayed high discounting and considered food to be highly reinforcing lost less weight (Best et al., 2012), whereas Manasse et al. (2017) did not find delay discounting to be predictive of weight loss during a standard behavioral treatment.

Thus, evidence linking the specific facets of executive function and delay discounting to BMI and weight loss is inconsistent. Further, few studies have included all three facets of executive function in a single study. Therefore, the aim of the current study was twofold. First, we aimed to clarify the relationships between each facet of executive function and BMI, by comparing executive function performance, self-reported executive function, and delay discounting between individuals with obesity and healthy weight controls. We hypothesized that individuals with obesity would show weaker performance, and report lower scores, on executive function tasks and measures, and display more delay discounting, relative to the healthy weight controls. The second aim of this study was to examine whether executive function and delay discounting would predict weight loss following a multidisciplinary weight loss intervention. We hypothesized that individual differences in executive function and delay discounting among individuals with obesity at the start of a weight loss treatment would predict changes in BMI, such that better inhibition, working memory and shifting, and less delay discounting, would be associated with more weight loss controlling for weight at baseline. Determining whether baseline executive function or delay discounting predicts treatment success would provide additional targets for intervention.

## Methods

### Participants

The sample of individuals with obesity were clients from an obesity treatment center who were about to start a multidisciplinary program targeting weight loss and lifestyle change (*n *= 82, 21 male, mean age = 41.12 ± 12.62 years, age range 18–71 years). All clients who completed their intake session at the center and were eligible to start the weight loss intervention were either personally approached by a research assistant or received a flyer with information about the study. The test-session was scheduled within 2 weeks of the start of the weight loss intervention. Participants gave written permission for researchers to access their weight loss data at the weight loss center. The healthy weight controls were recruited from the general population via advertisements. They were matched on a group level to the individuals with obesity on age, gender and education level (*n *= 71, 16 male, mean age = 43.39 ± 13.44 years, age range 18–69 years). See Table 1 for an overview of baseline characteristics of the sample. Groups did not differ in age, gender or education level (all *p*’s > .28), indicating that the matching procedure on a group level was successful. Individuals with obesity displayed, as expected, a higher BMI and a higher global Eating Disorder Examination-Questionnaire score, and they were strongly motivated to lose weight (all *p*’s< .001). Six participants who completed the baseline measurement and were included in the first analysis, dropped out during the multidisciplinary treatment. As there is no weight loss data available for these participants, the final sample for the prediction of weight loss (*n *= 76)^1^^,^^2^ included 18 (23.7%) males, with a mean age of 42.12 (*SD *= 12.47, range 19–71) and a mean BMI of 39.19 (*SD* = 5.35, range 30.90–65.38). The six participants who dropped out during treatment did not differ from the final sample on gender, education, motivation to lose weight or Eating Disorder Examination-Questionnaire score (all *p*’s > .13), though they were significantly younger (drop-out: *M* = 28.50, *SD *= 6.41. *p* < .01). The present study was approved by the local Ethical Review Commission.

**Table 1:** Characteristics of the sample displayed per weight group (*N* = 153)

	HWC (*n *= 71) *M* (*SD*)/%	OB (*n *= 82) *M* (*SD*)/%	*t* or *χ*^2^ (*df*)^a^	*p*
Age	43.40 (13.44)	41.12 (12.62)	1.08 (151)	.28
Gender			0.06 (1)	.80
Female	77.5%	74.4%		
Education level			0.48 (2)	.79
Low	11.3%	11.0%		
Medium	64.8%	69.5%		
High	23.9%	19.5%		
BMI	22.63 (1.53)	38.94 (5.24)	− 26.90 (96.65)	**< .001**
EDE-Q	0.64 (0.65)	2.86 (1.03)	− 16.17 (138.76)	**< .001**
Motivation to lose weight	1.97 (0.91)	4.48 (0.49)	− 20.87 (103.56)	**< .001**

Bold values indicate *p*-value < .05 was considered significant

*HWC* healthy weight controls, *OB* individuals with obesity, *BMI* Body Mass Index, *EDE*-*Q* Eating Disorder Examination-Questionnaire

^a^Degrees of freedom vary across *t* tests depending on violation of Levene’s test for equality of variances; degrees of freedom were adjusted accordingly

### The behavioral treatment program at the weight loss center

The multidisciplinary treatment consisted of a 6 or 12-month program targeting weight loss and lifestyle change, tailored to the individual’s needs and development. The program focused on physical activity (weekly group sport session and individual exercising at home) and nutritional advice to promote a healthy diet by a dietician, as well as counselling by a psychologist based on principles of cognitive behavioral therapy. See Aller and van Baak (2016) for a more extended description and evaluation of the obesity treatment program.

### Materials and measures

#### 2-Back task

The n-back task was administered to measure working memory, based on the 2-back task used by Boselie et al. (2016). Stimuli (i.e., letters) were presented one-by-one on a computer screen, and participants had to indicate whether each letter was the same as the letter that was presented two stimuli ago by pushing ‘yes’ or ‘no’ on the keyboard. Within one trial, a single letter was presented on the screen for 500 ms (ms), followed by a blank screen for 1500 ms. Participants started with a practice phase consisting of 30 trials, in which they received feedback after every response. Next, they completed the testing phase, consisting of 90 trials (30 target and 60 non-target letters). The main outcome variable was accuracy, which was the summation of correctly identified targets (hits) and non-targets (correct rejections). A higher accuracy displayed better working memory.

#### Stop-Signal Task

The Stop-Signal Task was used to measure inhibition (Logan et al., 1997). Both a general Stop-Signal Task (with X and O as go stimuli) and a food-specific Stop-Signal Task (with food pictures displayed in landscape or portrait orientation as go stimuli) were administered in counterbalanced order. Participants were instructed to respond as fast as possible to the go stimuli by pushing a left or right response key on the keyboard. However, if an auditory stop signal (beep) was presented, the participant had to inhibit his or her response and not push the key. During a go trial the letter or picture was presented for 1000 ms, preceded by a 500 ms fixation point. The delay between the go signal and the stop signal (go–stop delay) started at 250 ms and was subsequently adjusted by a tracking procedure. If the participant successfully inhibited the response, the go–stop delay was increased by 50 ms, and if the participant was not able to inhibit the response, the delay decreased by 50 ms. The dependent variable was the stop signal reaction time, which was calculated by subtracting the mean stop delay from the mean reaction time. A higher stop signal reaction time is indicative of less inhibitory control. Percentage of inhibited trials was verified afterwards. If a participant never stopped at a stop signal, and thus failed to comply with the instructions, no stop signal reaction time could be calculated.

#### Trail Making Test

The Trail Making Test was administered to assess shifting (Reitan, 1992). The Trail Making Test consists of two parts. In part A, participants had to connect 25 circles numbered from 1 to 25. In part B, the participants had to connect 24 circles numbered from 1 to 12 with letters from A to L in alternating order. When an error was made, the participant was instructed to return to the place where the error originated and to continue from there. The outcome variable was the difference score, which was the time to complete part B minus time to complete part A (B–A). This outcome measure provides a relatively pure indicator of shifting ability, as it minimizes visuoperceptual and working memory demands (Sanchez-Cubillo et al., 2009). The higher the difference score, the poorer the shifting ability.

#### Monetary Choice Questionnaire

Participants completed the 27-item Monetary Choice Questionnaire (Kirby et al., 1999) which is a common measure of delay discounting. They were presented with 27 hypothetical choices between smaller, immediate monetary rewards and larger, delayed monetary rewards. An example choice is ‘Would you prefer €55 today, or €75 in 61 days?’ Participants’ hyperbolic discount parameter (k) was determined for small, medium and large rewards (Kaplan et al., 2014). To reduce the number of variables in our analyses, the geometric mean of k was used as outcome variable. Values of k could range from 0.00016 to 0.25, with higher values indicating a preference for smaller, immediate rewards over larger, delayed rewards, thus a higher level of discounting. Because raw k values tend to be skewed, k values were normalized using a log transformation.

#### Behavioral Rating Inventory of Executive Functioning-Adult Version

The Behavioral Rating Inventory of Executive Functioning-Adult Version (Roth & Gioia, 2005) is a 75-item standardized rating scale developed to provide a window into everyday behaviors associated with specific domains of executive functioning in adults. The questionnaire consists of nine subscales, of which we used the three subscales which correspond to the three facets of executive function targeted in the current study: the working memory, inhibit, and shift subscales. Participants had to indicate for each item whether the statement applied to them on a 3-point Likert scale, with the options ‘never’, ‘sometimes’ or ‘always’. Example item are: ‘I tap my fingers or bounce my legs’ (inhibit), ‘I have trouble concentrating on tasks (such as chores, reading, or work)’ (working memory), ‘I have trouble changing from one activity or task to another’ (shift). The raw scale scores were transformed to T-scores (standardized scores with M = 50 and SD = 10, T ≥ 65 = clinically significant, Roth & Gioia, 2005) with higher T-values reflecting weaker executive function.

#### Eating Disorder Examination-Questionnaire 6.0

The Eating Disorder Examination-Questionnaire 6.0 (Fairburn & Beglin, 2008) is a 28-item self-report questionnaire that assesses disordered eating behaviors and attitudes over the previous 28 days, and was assessed to characterize the sample. Each item is scored on a 7-point scale indicating the frequency or severity of the item. The items are scored across four subscales: restraint, weight concerns, shape concerns, and eating concerns, and combined into one global score with higher scores reflecting greater levels of eating psychopathology.

#### Motivation to lose weight

Participants indicated on a 5-point Likert scale the extent of importance of four statements regarding motivation to lose weight, ranging from (1) ‘totally not important’ to (5) ‘extremely important’. An example of an item is: ‘How important is it for you to lose weight?’ The four items showed good reliability, with a Cronbach’s alpha of .96, and therefore a total score was calculated by summing up the scores of the individual items and taking the average, with a higher score indicating a stronger motivation to lose weight.

#### Percentage BMI loss (e.g. BMI change)

BMI data of individuals with obesity at baseline and after 6 months of treatment were provided by the obesity treatment center. Participants were weighed and measured while wearing their clothes but without shoes. BMI was calculated with the formula: (height in cm)/(weight in kg)^2^. To correct for initial differences in BMI between participants, change in BMI was calculated as a percentage of BMI loss relative to baseline BMI (Deitel & Greenstein, 2003). Percentage BMI loss was calculated with the formula: ((BMI baseline − BMI post-measurement)/BMI baseline) × 100.

### Procedure

Participants completed one individual session in which executive function and delay discounting were assessed via behavioral tasks and questionnaires. Individuals with obesity were tested at the obesity treatment center in a private room, whereas healthy weight controls were tested at the university. First, participants performed the behavioral tasks to measure executive function performance (2-back task, Trail Making Test, Stop-Signal Task-general and Stop-Signal Task-food). The behavioral tasks were administered in randomized order. Next, participants completed the Monetary Choice Questionnaire to measure delay discounting, the Behavioral Rating Inventory of Executive Functioning, Eating Disorder Examination-Questionnaire, motivation to lose weight and demographics. Finally, healthy weight controls were measured and weighed at the university. Individuals with obesity were measured and weighed at the obesity treatment center at the start of their weight loss intervention and again 6 months later. All participants received a voucher of €10 as compensation for participation.

### Statistical analyses

First, the data were inspected for outliers (i.e., mean ± 3 standard deviations). One outlier was observed for the Trail Making Test (healthy weight controls), and two outliers were observed for the Stop-Signal Task-general (individuals with obesity). These outliers were observed above the mean and were therefore adjusted to the highest value that was not considered an outlier plus one for each measure. To investigate the association between behavioral tasks and questionnaires, Pearson correlation coefficients were calculated. Next, individuals with obesity at baseline and healthy weight controls were compared on their performance on the executive function tasks and self-report measures using MANOVA’s testing for group differences in (1) working memory, general and food-specific inhibition, shifting and delay discounting, and (2) the working memory, inhibit and shift subscales of the Behavioral Rating Inventory of Executive Functioning (self-report). Significant effects were further analyzed using Bonferroni corrected univariate statistics. Further, hierarchical regression analyses were conducted with percentage BMI loss as outcome variable. Age, gender and education level were entered in step one. First we looked at the predictive effect of each measure by entering them individually into the model. Next, given that there was no a priori hypothesis about which factor would be most predictive, we performed two hierarchical regression analyses, one containing the behavioral measures including delay discounting and one containing the self-report measures. After entering demographics in step one, the other predictors were entered simultaneously in step two in a forced approach.

## Results

### Descriptives

See Table 2 for an overview of correlations between predictors. Behavioral working memory correlated moderately with all other predictors, indicating that better performance on the 2-back task was related to better performance on the other executive function tasks, less delay discounting and better self-reported executive function. The general and food-specific Stop-Signal Task displayed a strong positive correlation, indicating that both measures capture different, though related facets of behavioral inhibition. The behavioral measure of shifting was positively associated with self-reported shifting, though note that the correlation with self-reported working memory and inhibition was also significant and comparable to this result. Self-reported inhibition correlated with food-specific but not general behavioral inhibition. The Behavioral Rating Inventory of Executive Functioning subscales were moderate to strong positively correlated with each other.

**Table 2:** Zero-order correlations between all predictors (*N* = 153)

	Working memory-behavioral	General inhibition-behavioral	Food inhibition-behavioral	Shifting-behavioral	Delay discounting	working memory-self-report	Inhibition-self-report	Shifting-self-report
Working memory-behavioral	x							
General inhibition-behavioral	− .26**	x						
Food inhibition-behavioral	− .22**	.59***	x					
Shifting-behavioral	− .48***	.29***	.12	x				
Delay discounting	− .21*	− .01	.01	.04	x			
Working memory- self-report	− .19*	.18*	.23**	.21*	.03	x	.	
Inhibition-self-report	− .18*	.11	.17*	.18*	− .01	.57***	x	
Shifting-self-report	− .20*	.14	.11	.19*	.01	.70***	.44***	x

*p < .05; **p < .01; ***p < .001

### Comparing executive function between individuals with obesity and healthy weight controls

A one-way MANOVA was conducted to investigate group differences in executive function and delay discounting. Five dependent variables were included in the first MANOVA: behavioral working memory, behavioral shifting, general behavioral inhibition and food-specific behavioral inhibition and delay discounting (see footnote 1, 2). Overall, the MANOVA revealed a significant difference between groups, *F* (5,139) = 3.73, *p* < .01, η_*p*_^2^ = .12. Post-hoc analyses using a Bonferroni adjusted alpha level of .01, revealed significant differences between groups for both inhibitory control outcomes: general behavioral inhibition, *F* (1,143) = 13.18, *p* < .001, η_*p*_^2^ = .08, and food-specific behavioral inhibition, *F* (1,143) = 10.30, *p* < .01 η_*p*_^2^ = .07. Inspection of the mean scores indicated that individuals with obesity displayed a higher stop signal reaction time (i.e., less behavioral inhibition) on the general and food-specific Stop-Signal Task than healthy weight controls (see Table 3 for an overview of all means and standard deviations per group).

**Table 3:** Univariate follow-up analyses of comparison between weight groups, means with standard deviations in parentheses are displayed

Predictors	HWC (*n *= 71) *M* (*SD*)	OB (*n *= 82) *M* (*SD*)	*F* (*df*)^a^	*p*
Working memory-behavioral	72.30 (14.57)	72.83 (11.45)	0.06 (1, 143)	.81
General inhibition-behavioral	287.76 (50.15)	319.66 (55.29)	13.18 (1, 143)	**< .001**
Food inhibition-behavioral	329.39 (61.26)	347.51 (69.55)	10.30 (1, 143)	**< .01**
Shifting-behavioral	23.77 (14.81)	26.29 (14.45)	0.24 (1, 143)	.30
Delay discounting	− 4.77 (1.83)	− 4.62 (1.87)	1.08 (1, 143)	.63
Working memory-self report	53.52 (10.17)	59.35 (12.63)	9.70 (1, 151)	**< .001**
Inhibition-self report	51.01 (9.82)	55.22 (10.62)	6.40 (1, 151)	.**01**
Shifting-self-report	52.66 (9.09)	55.71 (11.32)	3.30 (1, 151)	.07

Applying a Bonferroni adjusted alpha level of .01 for the behavioral outcomes, a *p*-value < .01 indicated in bold was considered significant for the first five outcomes, and applying a Bonferroni adjusted alpha level of .017 for the self-report outcomes, a *p*-value < .017 indicated in bold was considered significant for the last three outcomes

*HWC* healthy weight controls, *OB* individuals with obesity

^a^Degrees of freedom vary due to missing data at random, as MANOVA applies listwise deletion

A MANOVA including the three subscales of the behavioral rating of executive functioning (working memory, inhibit and shift) was performed to investigate group differences in self-reported executive function. Overall, the MANOVA revealed a significant difference between groups, *F* (3, 149) = 3.61, *p* = .015, η_*p*_^2^ = .07. Post-hoc analyses, using a Bonferroni adjusted alpha level of .017, indicated that there were significant differences between groups on both self-reported working memory and inhibition: working memory, *F* (1,151) = 9.70, *p* = .002, η_*p*_^2^ = .06, and inhibition, *F* (1,151) = 6.40, *p* = .012, η_*p*_^2^ = .04. Results for shifting did not reach significance, *F* (1,151) = 3.30, *p* = .071, η_*p*_^2^ = .02. Inspection of the mean scores indicated that individuals with obesity reported higher T-values (e.g. weaker executive function) on the working memory, inhibition and shifting subscales (see Table 3).

### Executive function as predictor of BMI change

Participants lost on average 2.95 BMI points (*SD* = 1.98), though results ranged from an increase of 0.40 to a decrease of 10 BMI points. Regarding percentage BMI loss, participants lost on average 7.22% of their baseline BMI (*SD* = 4.80%), ranging from an increase of 1.16% in BMI to a decrease of 23.50%. In the hierarchical regression analysis predicting percentage BMI loss, demographics (age, gender and education level) were entered in step one, and each predictor was entered individually at step two. Demographics predicted a total of 7.5% of variance in percentage BMI loss. Results revealed behavioral working memory to be a significant predictor of BMI change (*β *= .31, *p *= .03), and a marginally significant contribution of self-reported inhibition was found (*β *= − .23, *p *= .051). None of the other predictors reached significance (all *p *> .39).

Subsequently, a hierarchical regression analysis was performed with all behavioral measures including delay discounting being entered at once in step two. Demographics predicted a total of 7.5% of variance in percentage BMI loss, and adding all behavioral predictors in step two accounted for an additional 8.5% of variance. The model as a whole did not reach significance, *F* (9, 61) = 1.29, *p* = .26. Table 4 shows the results of the hierarchical model including all behavioral measures. In step two, besides education and a marginally significant contribution of gender, behavioral working memory was a significant predictor of BMI change. This indicates that a better working memory is related to more weight loss. A final model containing gender, education and behavioral working memory did reach significance, *F* (4, 70) = 2.60, *p* = .04. After controlling for gender and education, behavioral working memory explained an additional 5.4% of variance, *F* change (1, 70) = 4.36, *p* = .04

**Table 4:** Results of hierarchical linear regression analyses predicting percentage Body Mass Index loss after 6 months of multidisciplinary treatment with behavioral measures (*n* = 76)

Fixed effect	*B*	*SE B*	*β*	t	*p*
Step 1					
Constant	9.77	2.74			
Age	0.00	0.05	.01	0.05	.96
Gender	− 2.47	1.36	− .22	− 1.82	.07
Medium education	− 0.47	1.79	− .05	− 0.26	.80
High education	− 2.07	2.07	− .18	− 1.00	.32
Step 2					
Constant	− 2.46	7.28			
Age	0.01	0.05	.01	0.11	.91
Gender	− 2.63	1.52	− .24	− 1.74	.09
Medium education	− 2.78	2.08	− .27	− 1.34	.19
High education	− 5.19	2.55	− .44	− 2.04	**.05**
Working memory-behavioral	0.15	0.07	.37	2.27	**.03**
General inhibition-behavioral	0.00	0.01	.01	0.09	.93
Food inhibition-behavioral	0.00	0.01	.04	0.23	.82
Shifting-behavioral	0.03	0.05	.09	0.57	.57
Delay discounting	− 0.27	0.33	− .10	− 0.81	.42

Bold values indicate *p*-value < .05 was considered significant

*R*^2^= .08 for Step 1, Δ*R*^2^= .09 for Step 2 (*p *= .30)

Next, a hierarchical regression analysis was conducted with all self-report measures entered in step two. This accounted for an additional 10% of variance. The model as a whole was marginally significant, *F* (7, 68) = 1.99, *p* = .07. Table 5 shows the results of the hierarchical model including all self-report measures. Self-reported inhibition was a significant predictor in step two, besides gender, indicating that self-reported inhibition explains a significant part of variance of percentage BMI loss. A final model containing gender and self-reported inhibition did reach significance, *F* (2, 74) = 3.56, *p* = .03. After controlling for gender, inhibition explained an additional 3.5% of variance, *F* change (1, 74) = 2.81, *p* = .098.

**Table 5:** Results of hierarchical linear regression analyses predicting percentage Body Mass Index loss after 6 months of multidisciplinary treatment with self-report executive functioning (*n* = 76)

Fixed effect	*B*	*SE B*	*β*	t	*p*
Step 1					
Constant	9.77	2.64			
Age	0.00	0.05	.01	0.05	.96
Gender	− 2.47	1.31	− .22	− 1.89	.06
Medium education	− 0.47	1.73	− .05	− 0.27	.79
High education	− 2.07	1.99	− .18	− 1.04	.30
Step 2					
Constant	− 14.79	4.63			
Age	− 0.03	0.05	− .07	− 0.58	.57
Gender	− 3.22	1.32	− .29	− 2.44	**.02**
Medium education	− 1.37	1.71	− .13	− 0.80	.43
High education	− 2.24	2.00	− .19	− 1.12	.27
Working memory-self-report	0.06	0.07	.17	0.95	.35
Inhibition-self-report	− 0.17	0.06	− .37	− 2.71	.**01**
Shifting-self-report	0.05	0.07	.11	0.65	.52

Bold values indicate *p*-value < .05 was considered significant

*R*^2^= .08 for Step 1, Δ*R*^2^= .10 for Step 2 (*p *= .06)

## Discussion

The main aim of this study was twofold: (1) to examine whether individuals with obesity would show weaker performance on executive function measures and display more delay discounting relative to healthy weight controls, matched (group level) on age, gender and education level, and (2) whether executive function and delay discounting would be predictive of weight loss during a subsequent multidisciplinary weight loss treatment. This study included both behavioral and self-reported measures of the three main facets of executive function (Diamond, 2013; Miyake et al., 2000) and delay discounting in one study. Results show that individuals with obesity displayed less efficient general and food-specific behavioral inhibition, relative to healthy weight controls and that they reported weaker executive functioning in daily life. Individuals with obesity did not display weaker behavioral working memory or shifting than healthy weight controls. Regarding the prediction of weight loss, behavioral working memory was the strongest predictor of change in BMI, besides gender and education level. In addition, more difficulties in daily life with respect to inhibition as indicated on the inhibit subscale of the Behavioral Rating Inventory of Executive Functioning contributed marginally significantly to the prediction of BMI loss.

Performance on both the general and food-specific Stop-Signal Task differed between individuals with obesity and healthy weight controls, with individuals with obesity displaying less efficient inhibition. These results are consistent with those of Bartholdy et al. (2016); however, they differ from Houben et al. (2014), who only found an association between elevated BMI and weaker performance on a food-specific Stop-Signal Task. The inconsistency in findings may be explained by the different samples used in each study. The current sample included individuals with obesity rather than predominantly individuals who were overweight. It may be the case that differences in general inhibition are only apparent at the higher end of the weight spectrum. Previous research did show promising results of food-specific inhibition training on food intake and weight loss (Houben & Jansen, 2015; Lawrence et al., 2015), whereas studies in which general inhibition was targeted were not successful (Allom et al., 2016a).

Individuals with obesity reported more problems experienced in daily life as indicated on the Behavioral Rating Inventory of Executive Functioning (though note that self-reported shifting did not reach significance), in line with our hypothesis. While weaker working memory was reported by individuals with obesity, behavioral working memory as measured with the 2-back task did not differ between the weight groups. Behavioral tasks and ratings of executive functioning have been suggested to reflect different underlying constructs (Allom et al., 2016b; Toplak et al., 2013). Thus, based on the current results, obesity is not associated with working memory and shifting as measured by behavioral tasks, though individuals with obesity do seem to experience less successful goal pursuit in daily life.

The current results indicate that behavioral inhibition and self-reported executive function are important in preventing obesity, as they appear to be a risk factor for weight gain (e.g. Dohle et al., 2018; Hofmann et al., 2008). However, it is uncertain whether these factors indeed cause obesity, or obesity causes poorer executive function, or whether there is another factor explaining both. There are indications that impaired executive function leads to impaired self-regulation (e.g. higher food intake, less exercise), rendering individuals with deficient executive function more predisposed to becoming obese (Dohle et al., 2018; Hofmann et al., 2012). However, there is also evidence that obesity leads to impaired cognitive functioning via reduced blood flow to the areas of the brain that control executive function or abnormalities in glucose and insulin regulation (Boeka & Lokken, 2008; Smith et al., 2011). Conversely, the results of a recent meta-analysis indicated a significant positive effect of weight loss on executive function (Veronese et al., 2017). This suggests that the association between executive function and obesity is bidirectional (Kanoski & Davidson, 2011; Sellbom & Gunstad, 2012). To shed more light on this relationship, future research should study these factors in prospective designs including pre- and posttests or experimental designs including a manipulation of executive function.

Rather than focusing on weight gain, the present study focused on examining the prediction of weight loss. Behavioral working memory was the strongest predictor of a decreased BMI after treatment, suggesting an important role of working memory in weight loss success. Specifically, working memory may help to keep weight loss goals in an active state, so someone can efficiently monitor whether their food intake is in line with their weight loss goals (Boutelle & Kirschenbaum, 1998). This is in line with dual process models that suggest that the relative influence of each system on self-regulation differs as a function of working memory such that individuals with better working memory display more goal-directed behavior (e.g. Hofmann et al., 2008). Our results are in line with Hege et al. (2013), who showed the predictive value of behavioral working memory for successful weight loss during a lifestyle treatment. Hege et al. (2013), however, used a food-specific working memory task, which could be even more predictive in the self-regulation of weight loss given the role of working memory in food cue monitoring (Meule, 2016). Based on these results, targeting weaker working memory seems a promising target for intervention (Jansen et al., 2015). Preliminary results of working memory training indicate short-term effects of training on food intake, though long-term effects on weight loss have not yet been established (Dassen et al., 2018; Houben et al., 2016).

Self-reported inhibition was a significant predictor of weight loss, with participants who reported less efficient inhibition in daily life displaying a smaller BMI change. The behavioral measures (e.g. general and food-specific Stop-Signal Task), in contrast, were not related to weight loss. This is contrary to our hypothesis and not in line with previous research (e.g. Galioto et al., 2016; Manasse et al., 2017; Nederkoorn et al., 2007). Shifting as measured with the Trail Making Test did not differ between individuals with obesity and healthy weight controls, and was also not predictive of subsequent weight loss. Importantly, many executive function tasks were originally developed as indicators of brain damage, including the Trail Making Test (Reitan, 1958). Therefore, it is possible that this task was not sensitive enough to pick up subtle impairments in shifting ability (Fitzpatrick et al., 2013). Task impurity is also a common problem in this field of research, as tasks may be thought to measure a particular facet of executive function but the execution of any task may also involve diverse cognitive systems in addition to the targeted facet (Jurado & Rosselli, 2007). The role of shifting in eating behavior is relatively unexplored (Dohle et al., 2018). Shifting ability may be beneficial to weight loss, helping individuals to switch strategy when the current strategy is suboptimal, though this ability could also allow individuals to easily switch from their weight loss goal to more tempting immediate options (Hofmann et al., 2012). However, in the current study, no association of behavioral or self-reported shifting with weight loss was found.

Delay discounting did not differ between individuals with obesity and healthy weight controls, and also did not add significantly to the prediction of weight loss, though results were in the expected direction. In the current study, we measured delay discounting with the Monetary Choice Questionnaire, which uses money as a reinforcer. However, it has been suggested that a food-specific delay discounting task would be more predictive of BMI and weight loss (e.g. Rasmussen et al., 2010). Current results provide no evidence for a direct relationship of delay discounting with weight loss. Importantly, we followed participants until 6 months after the start of their weight loss intervention. As weight regain is one of the biggest problems in weight loss treatment (Ikeda et al., 2005), it would be interesting to continue to monitor participants after finishing the treatment, to examine the potential role of executive function and delay discounting in the maintenance of weight loss (Gettens & Gorin, 2017). It has been suggested that self-regulation becomes even more important when the intensive treatment ends, as the external regulation coming from the treatment program also ends at this point (Halberstadt et al., 2013).

In conclusion, executive function appears to be weaker in individuals with obesity, in line with the results of a recently published meta-analysis (Yang et al., 2018). We discovered weaknesses on self-report measures and both general and food-specific behavioral inhibition. Executive function is also predictive of weight loss, with behavioral working memory being associated with a greater decrease in BMI following treatment, and a marginally significant contribution of self-reported inhibition was found. A significant contribution of shifting or delay discounting to the prediction of weight loss could not be established. Thus, it seems that differences in some facets of executive function between individuals with obesity and healthy weight individuals exist, but other facets of executive function seem responsible for weight loss success. Based on current results, behavioral inhibition can differentiate between obese and healthy weight individuals, while behavioral working memory predicts weight loss. Thus, the impact of specific executive functions may differ between different weight-related behaviors (Gettens & Gorin, 2017), and future research should distinguish between the role of each facet of executive function in weight gain and weight loss, as this would have different implications for which facets of executive function are important for prevention of obesity, and which facets would be important for intervention. Based on the current results, it would be interesting for future studies to explore whether adding a cognitive screening at the start of a weight loss trajectory could be an objective way to identify individuals who might benefit from additional executive function training to facilitate optimal weight. These results suggest new avenues for the improvement of current behavioral treatments, and may increase future treatment success.

## Footnotes

1 Two data points (individuals with obesity) and one additional value (healthy weight controls) were excluded from the general Stop-Signal Task because the percentage of correct stop trials was zero, indicating that these participants never inhibited their response. Two additional data points (individuals with obesity) were excluded from the food-specific Stop-Signal Task because the percentage of correct stop trials was zero.

2 One participant was excluded from all behavioral tasks because of a physical disability affecting reaction times (individuals with obesity); one participant was excluded from the Trail Making Test because of wrong execution of instructions (individuals with obesity); data is missing for one participants for both the general and food-specific Stop-Signal Task because of technical problems in data storage (individuals with obesity); data is missing for one participant on the 2-back task because the participant did not correctly understand the instructions (individuals with obesity).
